# Health care provider knowledge and routine management of pre-eclampsia in Pakistan

**DOI:** 10.1186/s12978-016-0215-z

**Published:** 2016-09-30

**Authors:** Sana Sheikh, Rahat Najam Qureshi, Asif Raza Khowaja, Rehana Salam, Marianne Vidler, Diane Sawchuck, Peter von Dadelszen, Shujat Zaidi, Zulfiqar Bhutta, the CLIP Working Group, Payne Beth, Aina Olabisi, Chomiak Marianne, Olukayode A. Dada, Drebit Sharla, Firoz Tabassum, Goudar Shivaprasad, Kariya Chirag, Katageri Geetanjali, Lee Tang, Li Jing, Lui Man Sun, Makanga Tatenda, Ramadurg Umesh, Sharma Sumedha, Solarin Kunle, Laura A. Magee

**Affiliations:** 1 Division of Women & Child Health, Aga Khan University, Karachi, Pakistan; 2 Department of Obstetrics and Gynaecology, and the Child and Family Research Institute, University of British Columbia, Vancouver, V5Z 4H4 Canada; 3 Department of Research, Vancouver Island Health Authority, Victoria, V8R 1J8 Canada; 4 Department of Obstetrics and Gynaecology, St George’s, University of London, London, SW17 0RE UK; 5 Program for Global Pediatric Research, Hospital for Sick Children, Toronto, M5G 2L3 Canada

**Keywords:** Community health services, Eclampsia, Health personnel, Pre-eclampsia, Community health worker, Pregnancy, Pakistan

## Abstract

**Background:**

Maternal mortality ratio is 276 per 100,000 live births in Pakistan. Eclampsia is responsible for one in every ten maternal deaths despite the fact that management of this disease is inexpensive and has been available for decades. Many studies have shown that health care providers in low and middle-income countries have limited training to manage patients with eclampsia. Hence, we aimed to explore the knowledge of different cadres of health care providers regarding aetiology, diagnosis and treatment of pre-eclampsia and eclampsia and current management practices.

**Methods:**

We conducted a mixed method study in the districts of Hyderabad and Matiari in Sindh province, Pakistan. Focus group discussions and interviews were conducted with community health care providers, which included Lady Health Workers and their supervisors; traditional birth attendants and facility care providers. In total seven focus groups and 26 interviews were conducted. NVivo 10 was used for analysis and emerging themes and sub-themes were drawn.

**Results:**

All participants were providing care for pregnant women for more than a decade except one traditional birth attendant and two doctors. The most common cause of pre-eclampsia mentioned by community health care providers was stress of daily life: the burden of care giving, physical workload, short birth spacing and financial constraints. All health care provider groups except traditional birth attendants correctly identified the signs, symptoms, and complications of pre-eclampsia and eclampsia and were referring such women to tertiary health facilities. Only doctors were aware that magnesium sulphate is recommended for eclampsia management and prevention; however, they expressed fears regarding its use at first and secondary level health facilities.

**Conclusion:**

This study found several gaps in knowledge regarding aetiology, diagnosis and treatment of pre-eclampsia among health care providers in Sindh. Findings suggest that lesser knowledge regarding management of pre-eclampsia is due to lack of refresher trainings and written guidelines for management of pre-eclampsia and presentation of fewer pre-eclamptic patients at first and secondary level health care facilities. We suggest to include management of pre-eclampsia in regular trainings of health care providers and to provide management protocols at all health facilities.

**Trial registration:**

NCT01911494

**Electronic supplementary material:**

The online version of this article (doi:10.1186/s12978-016-0215-z) contains supplementary material, which is available to authorized users.

## Plain english summary

High blood pressure in pregnancy is one of the major three causes of maternal deaths in Pakistan. Different research studies have shown that health care providers have limited knowledge regarding management of these patients. In this study we explored knowledge of community and hospital based health care providers regarding causes, complications and treatment of the disease. Doctors, Lady Health Workers, Lady Health Supervisors and traditional birth attendants were interviewed in groups and one to one in Sindh province. Along with interviews Lady Health Workers were also asked to fill a questionnaire to assess their competency and knowledge. Study found that community based health care providers consider stress and physical work load a cause of high blood pressure. Only doctors were aware of the first line medicine to treat severe cases of high blood pressure in pregnancy but they were concerned regarding the safety of drug to be used in small health facilities. Regular trainings of care providers are required to improve their knowledge and practices to deal with pregnant women with high blood pressure.

## Background

Pre-eclampsia is defined as development of new hypertension in pregnancy along with significant proteinuria occurring after 20 weeks of gestation [1]. It is a multisystem disorder that may affect the liver, kidney and clotting in pregnancy, as well as potential fetal growth restriction and premature delivery [1]. Eclampsia is a complication of pre-eclampsia defined as the new onset of grand mal seizure(s) and/or unexplained coma during pregnancy or postpartum in a woman with pre-eclampsia [2]. Eclampsia is responsible for one in ten maternal deaths, and claims 2000 maternal lives every year, in Pakistan [3, 4].

Several multicounty trials [5, 6] and systematic reviews [7] have proved that MgSO_4_ is an important agent in the management of severe pre-eclampsia and eclampsia. The World Health Organization (WHO) stated that MgSO_4_ is the first line drug for treatment of pre-eclampsia and eclampsia more than a decade ago [8] and Pakistan included MgSO_4_ in the national essential drug list in 2007 [9]. However, these efforts have not translated into practice and a large number of women continue to suffer from pre-eclampsia and eclampsia without receiving life-saving treatment.

In Pakistan, the health care system is comprised of both formal and informal sectors. Formal system included public and private health facilities. Pakistan’s public health system is centralised under the Federal Government and Provincial Health Ministries; and comprises of primary, secondary and tertiary health centres. Primary care facilities include 5000 Basic Health Units, 560 Rural Health Centres, 900 Maternal and Child Health centres and large number of dispensaries and first aid posts. Secondary level centres include 900 Taluka and district level hospitals. Tertiary health care is delivered through 30 teaching hospitals [10].

Lady Health Workers (LHWs) and Lady Health Visitors (LHVs) are deployed as community-based health care workers in the home and primary health centres [10]. Doctors and nurses are deployed at all levels of health care facilities. The private sector consists of a few accredited tertiary level hospitals and a large number of non-accredited tertiary, secondary and primary clinics and hospitals both in urban and rural areas. The informal health care system is led by non-certified local health care providers, such as traditional birth attendants (TBA), spiritual healers, and *Hakeems (practitioner of Unani/Greek medicine).* The informal health care system is patronised by many as treatment is affordable, available within the local community, and in-line with traditional and cultural beliefs [11].

Studies from other low and middle-income countries (LMIC) reported that contrary to WHO guidelines, women were not regularly screened for high blood pressure during antenatal care [12]. Literature from the developing world has also reported that various cadres of health care providers (doctors, nurses, midwife, and community care providers) have limited knowledge regarding screening and management of pre-eclampsia [13–15].

The limited knowledge of health care providers likely plays a role in the slow reduction in maternal morbidity and mortality due to pre-eclampsia in developing countries, such as Pakistan. Hence, we explored the knowledge of different health care providers regarding pre-eclampsia and eclampsia and their current management practices in rural Sindh, Pakistan.

### Study area

This study was conducted in Matiari and Hyderabad, which are two districts of Sindh province. Sindhi is the most common language of both districts. Hyderabad has a population of 4.5 million of which 60 % live in urban areas, it is second most urbanised district of Sindh after Karachi [16]. Matiari district is located 250 km north of Karachi, with a population of 0.6 million. The area is largely agricultural and development indicators are representative of rural Sindh [17] (Table 1).

**Table 1:** Comparison of population characteristics of Sindh Province with country estimates

Population characteristics	Provincial estimates [28]	National estimates [5]
% of women received skilled antenatal care	78	73
% of deliveries attended by skilled birth attendant	60	52
% of population covered by Lady Health Workers	45 [29]	65 [30]

## Methods

The Community Level Intervention for Pre-eclampsia (CLIP) study is a cluster randomized trial which is being conducted in two districts of Sindh Pakistan (NCT01911494). Before implementation of the trial, a feasibility study was conducted to evaluate barriers and facilitators of providing emergency treatment in community to women who are at high risk of severe pre-eclampsia. Lady Health Workers were chosen to deliver this intervention to pregnant women as they are responsible to provide antenatal care at home in rural areas. Intervention package includes triage and screening of pregnant women for risk of severe PE/E, administering oral antihypertensive and MgSO4 (if required) and referral. It was important to understand the existing practices and knowledge of health care providers regarding PE/E. We evaluated health care providers involved in maternal care whether they were community based (LHW, LHS, TBAs) or health facility based (Doctors). The feasibility assessment utilized mixed methods [18]. and intervention implementation plan was modified according to the findings of the feasibility study.

### Qualitative component

The qualitative research team organised the conduct of focus groups and interviews. Gender specific staff was allocated with respect of local culture and tradition. Project staff were locally recruited and trained by a senior faculty and a social scientist with first-hand knowledge and expertise in qualitative research. Each focus group was conducted in the local language by one facilitator, two note takers, an observer and all discussions were audio-recorded. Observers documented field notes to capture verbal and non-verbal communication in support of the documented text. In addition, the facilitators completed a self-reflection after each session to describe their thoughts and impressions to better contextualize the data, as well as, to protect against self-bias. Quality control was ensured through random observation of focus groups by the field co-ordinator, and an audit-trail of 20 % of transcripts. The audit-trail process included verifying content of transcripts with audio-recordings, and bi-weekly debriefing sessions with moderators and transcribers. The field staff then transcribed the data in Sindhi based on the audio recordings. The qualitative data was analyzed in Sindhi using NVivo version 10 [QSR, Doncaster Vic, Australia] to develop the themes and subthemes from an ethnographic approach.

The desired number of FGDs and KIIs were determined through response saturation and data collection was stopped when data saturation was reached at each site. Data Saturation was reached after 26 in-depth interviews and seven focus groups discussions. Participants were eligible if they consented to participate in study and were available for at least 60 min for the interview or discussion (Table 2).

**Table 2:** Health care provider interviews and focus groups

Health care providers	Method	Total number of interviews/discussions	Total number of participants
Women Medical Officer/Gynaecologist	Interview	9	9
Lady Health Supervisor	Interview	10	10
Traditional Birth Attendant	Interview	7	7
Lady Health Worker	Focus Group	7	64

### Quantitative component

Eligible participants were identified with the help of local community health workers and research medical officers during health facility visits. Written consent was obtained from all participants prior to data collection.

A self-administered questionnaire was completed by 457 LHWs, all questions were designed using a Likert scale (strongly disagree to strongly agreeThe knowledge of LHWs regarding pre-eclampsia was evaluated by a set of questions pertaining to their ability to identify danger signs of hypertension and seizures in pregnancy. Their current practice of referral and skills to administer drugs were also evaluated.

This study received ethical approval from Ethics Review Committee of Aga Khan University, Karachi, Pakistan; National Bioethics Committee of Pakistan and Institutional Review Board of University of British Columbia, Vancouver Canada.

## Results

Among health care providers who participated, all were providing care to 3–5 pregnant women daily for more than a decade, except one TBA and two doctors. Six out of nine women medical officers (WMO) were providing care at the primary and secondary level public health facilities. About 6 pre-eclampsia or eclampsia patients (range 0–100 in last 12 months) were received by (WMO) in last 12 months. TBAs did not report encountering any women with pre-eclampsia in the last year. According to Lady Health Supervisors (LHS) on average 3 women with pre-eclampsia are reported to them per year by LHWs.

### Knowledge regarding pre-eclampsia aetiology and consequences

Health care providers of all cadres reported that a normotensive woman can develop hypertension later in pregnancy which can lead to problems for mother and baby. Common reasons for pre-eclampsia mentioned by community-based health care providers (LHW, LHS and TBAs) were stress of daily life which included burden of care giving, physical workload, short birth spacing and financial stresses.

*“She looks after the children and again she gets pregnant. She has no such stamina (strength) to deal with the problems and that is the reason she also gets irritated, sometimes because of children, sometimes because of family problems and sometimes cause is financial problems. She starts thinking lot and her B.P starts to rise up”. (Focus group with LHWs)*

In addition, anaemia was mentioned as a risk factor by almost all community-based health care providers. All participant groups, except TBAs, correctly identified complications of pre-eclampsia: antepartum haemorrhage, premature delivery, stroke, brain haemorrhage, decreased fetal movements, and restricted fetal growth. Doctors, LHWs and their supervisors were aware that seizures occur as a consequence of hypertension. Similar result was found through survey of LHWs where 84.5 % (386/457) LHWs strongly agreed that seizure is a danger sign of pregnancy and 83.6 % (382) strongly agreed for bleeding as a danger sign. See Additional file 1: Table S2. TBAs were not aware of other harmful effects of disease and considered seizures to be directly related to stress and physical weakness.

*“One cause of seizures is that they are pregnant and the other cause is that she is anemic and stressed out.”(Interview with a TBA)*

All health care providers were aware that pre-eclampsia could result in miscarriage or death.

*“When blood pressure increases firstly it is dangerous for the mother, the mother can also die, even the baby can die, it can affect the mother in anyway, she can have paralysis, because of high blood pressure paralysis can occur.” (Focus group with LHWs)*

### Diagnosis and referral of pre-eclampsia

Knowledge for identifying a patient with pre-eclampsia varied greatly among different set of care providers. Doctors were the most knowledgeable group. They were aware that hypertension and proteinuria constitutes pre-eclampsia and these women require regular monitoring.

*“If the woman is pre-eclamptic, we will tell them to visit us every 15 days. She should get her blood pressure checked and she should take medicines on time.”(Interview with a WMO)*

Doctors working at first and second level health facility reported refering patients to tertiary facilities The reasons for referral of these patients included limited availability of support staff (nurses, midwives, LHVs), monitoring equipment and medicines required to manage severe pre-eclampsia or eclampsia.

*“When she comes with eclampsia and if she is having seizures then we refer her because in our health centre we don’t have facility for C-section. If she is not in a good condition and is having seizures, we try to get her delivery done soon as possible. If she is not able to have normal vaginal delivery then we refer her.”(Interview with a WMO)*

LHWs and LHSs mentioned that they refer patients to health facilities if there are any danger signs during pregnancy. Pregnant women are also reportedly referred if there is sudden increase in weight or oedema, reduced fetal movements, vertigo or blurred vision. This finding was supported by quantitative assessment of LHWs where 42.9 % (96) reported receiving trainings to identify complications in pregnancy and 51 % (233) mentioned training to refer and manage these patients. Many LHWs (79 %; 361) reported referring pregnant women in their routine practice. See Additional file 1: Tables S2 and S3.

*“Vomiting more than 3 months, slowly oedema starts, headache, having fever, bleeding during pregnancy then in that condition we refer to heath facility.” (Focus group with LHWs)*

Focus groups with TBAs revealed that the elder TBAs were critical of orthodox medicines and vaccines, and consider them harmful in pregnancy. Some of these TBAs mentioned that they referred women for vertigo or pain, but that this is due malnutrition and anaemia. Few young TBAs participated, though they appeared to be more aware of the serious consequences of hypertension in pregnancy and the importance of early referral in such cases.

*“We tell them that you must go for monthly or fortnightly check-ups to any good health facility which is in vicinity; go there and get your check-up.” (Interview with a TBA)*

TBAs commonly referred pregnant women when they consider vaginal delivery will be difficult (e.g. cephalo-pelvic disproportion, breech presentation) or when complications occur during delivery (obstructed labour, postpartum haemorrhage, decreased fetal movements). They also referred women for seizures; this was done because they believed it delays descent/delivery of baby. It was apparent from their responses that TBAs were not aware of the signs and symptoms of pre-eclampsia, and so reluctant to refer patients unless safe delivery at home is not possible,

*“Bone is short or large; (delivery) path is clear or not. Then we refuse to take case and advise them to get her to the hospital.”*

“*When we see that the fetal movements have stopped (in woman having fits), I ask the attendants to arrange the car quickly”. (Interview with a TBA)*

When referral is required, TBAs preferred private facilities because they are perceived to be better equipped. On the other hand, LHWs referred women to nearest health facility.

*“We will tell them to take her to the nearest hospital or clinic as soon as possible.” (Focus group with LHWs)*

### Management of pre-eclampsia

This study found huge gaps in the knowledge of treatment for pre-eclampsia among all cadres of health care providers. Doctors were aware of use of antihypertensive drugs for hypertension and MgSO_4_ as the first line for eclampsia but there were several misperceptions regarding the use of MgSO_4_. The most common misconception was fears of MgSO_4_ toxicity and the need for an intensive care unit for these women. The findings showed that doctors did not recognise the role of MgSO_4_ in reducing risk of morbidity and mortality if administered earlier. Hence patients are referred to a higher facility untreated or diazepam was given to control seizures. Doctors also reported that there was no guidelines available treatment of pre-eclampsia, except one doctor who was working in a private secondary facility.

*“No (we do not use), because it (MgSO*_*4*_ *) is not used at dispensary level.”*

*“MgSO*_*4*_ *we don’t give here because we need a separate doctor to manage it.”*

*“We monitor the blood pressure, and try to refer the eclamptic woman to the Civil Hospital as early as possible.” (Interview with a WMO)*

All doctors except one senior doctor, with greater than 25 years of experience, expressed a desire to learn more regarding the management of pre-eclampsia and argued there was a need for related guidelines. Participants stated that they infrequently encounter pre-eclampsia; therefore, they are not confident in managing these cases.

Among community-based health care providers the knowledge was increasingly limited. None of the TBAs and very few LHWs had ever heard of MgSO_4_. All LHWs were aware that hypertension and seizures need medical treatment and women should seek care at health facility but could not name any medications which should be given. However, 131 (28.7 %) LHWs reported that they can measure BP and 30 (6.6 %) had a BP device. See Additional file 1: Table S3. TBAs could not name antihypertensive medications, they could, however, describe their appearance (shape, colour or brand logo). TBAs mentioned that they do not treat hypertensive women themselves nor do they prescribe any medication. However, they mentioned giving some of the medicines themselves which they might have learnt over time from interaction with doctors. In addition to medicine low salt and low fat diet was advised by almost all TBAs and LHWs.

*“We give it (Aldomet) daily if BP is high. We advise it twice a day or daily at night. The patient must eat daily so that the patient’s blood pressure remains normal.” (Interview with a TBA)*

Only one TBA, who had 50 years of experience, reported giving alternative or traditional medicines (*phakki*) to control blood pressure.

*“We give 200, 250, grams Phakki, one spoon daily morning she is advised to take.” (Interview with a TBA)*

Similar to doctors community-based providers have seen few patients with eclampsia in their practice; therefore, they were willing to learn more about the condition, its risk factors, prevention and treatment.

## Discussion

This study found gaps in knowledge among community health care providers regarding causes of PE/E and its management. Health facility based care providers were aware of the etiology and complications of the disease but there were misperceptions and limited knowledge about use of Magnesium sulphate.

Studies have reported a common community perception that seizures of eclampsia are caused by supernatural forces [19] and the frequent use of herbal medicine delays health care seeking [20]. Community based care providers may not connect eclampsia and hypertension; however, none claimed to believe in evil charms or supernatural causes for the seizures and use of alternate medicine to treat eclampsia.

Pre-eclampsia is a serious condition, but because of the relatively low prevalence of the disease care providers had infrequent experience with these patients. This lack of exposure results in a reported lack of confidence in dealing with these patients. Pakistan has included current global management principles of pre-eclampsia in skilled birth attendants and nurses trainings [21], but in this study health care providers expressed desire for further training as they found current trainings insufficient. This finding is not consistent with Bigdeli’s findings from Pakistan that health care providers were satisfied with their training [14].

Data from other LMIC showed a preference for diazepam in cases of eclampsia [22] but in this study only two of the nine doctors mentioned regular use of diazepam. Despite prolific myths and fears related to MgSO_4_, no other drugs were preferred for treating eclampsia by the providers interviewed. This finding is consistent with another study on use of MgSO_4_ from Pakistan [14]. The preference for MgSO_4_ is very encouraging as use of diazepam in eclampsia is harmful for both mother and baby. Health care providers in this study commonly reported referring women with pre-eclampsia and eclampsia prior to the administration of lifesaving treatment. Similar finding has been reported in literature [14]. Explanations for this practice in Pakistan and elsewhere were related to the misperception that MgSO_4_ should only be used in tertiary level facilities [23]. Other reasons MgSO_4_ was not administered were the lack of written treatment and referral protocols. The lack of clear guidelines to manage pre-eclampsia has been reported earlier as a barrier to health care provision at first and secondary level [22].

Other studies have also found that health care providers at high level facilities had better knowledge of pre-eclampsia [24, 25]. This might be due to more exposure of such cases and the availability of written protocols.

### Strengths and limitations

Several studies have been published on barriers and facilitators to use MgSO4 in LMICs [14, 15, 26, 27]. This study explores significant aspects of management of pre eclampsia with respect to knowledge regarding disease, procedural ability, preference for use of drugs and clinical decision making of both skilled and unskilled care providers. Qualitative methods gave an insight to the misperceptions related to the condition and its treatment as health care providers were free to express their beliefs, fears and experiences. TBAs are preferred birth attendants for many rural women so it was important to evaluate their level of understanding for the disease. So far literature published from Pakistan on PE/E management only assessed practice of skilled care providers. Limitation of the study is that it did not inquire participants about management for severe pre-eclampsia and eclampsia separately therefore the study cannot determine the knowledge of health care providers for the prophylactic use of MgSO_4_ in severe pre-eclampsia. Future researches should explore ways to develop educational strategy and simple tools to empower first and second level health care providers to be able to approach obstetrical problems. This will help to expedite referral in rural pregnant women and initiating treatment in homes in severe cases which require urgent management even before referral. The curriculum for health care providers who are involved with pregnant women in communities should be reviewed and training should ensure acquisition of competency for dealing with obstetrical emergencies.

## Conclusion

This study reveals that even in presence of a national policy supporting pre-eclampsia programmes, providers lack confidence and/or competence in treating these women. Gaps in the knowledge of aetiology, diagnosis and treatment of pre-eclampsia among all cadres of health care providers were seen. Findings suggest that limited exposure to pre-eclampsia cases, the lack of refresher trainings and no written guidelines for management of the disease are important factors leading to inadequate knowledge. We suggest inclusion of management of pre-eclampsia in regular training of all health care providers and to provide management protocols at all levels of health care. There is also need for strong advocacy for use of MgSO_4_ as emergency treatment before referral from first and secondary level health facilities for the best maternal and fetal outcomes.

## Additional files

Additional file 1:
**Table S1.** Baseline attributes of health care providers, **Table S2.** LHWs competency and skills to provide maternal health care, **Table S3.** Administrative and logistic support available to LHW to provide maternal care. (DOC 44 kb)
Additional file 2: Review reports. (PDF 431 kb)

## Abbreviations

CLIP: Community level intervention for pre-eclampsia
LHS: Lady health supervisor
LHVs: Lady health visitors
LHWs: Lady health workers
LMIC: Low and middle-income countries
TBA: Traditional birth attendants
WHO: World Health Organization
WMO: Women medical officer
