# Supplementary material for: Health care provider knowledge and routine management of pre-eclampsia in Pakistan
Source: Reprod Health. 2016 Sep 30;13(Suppl 2):104. doi: 10.1186/s12978-016-0215-z (PMC5056497; doi:10.1186/s12978-016-0215-z)
Supplement: Additional file 1: — Table S1. Baseline attributes of health care providers, Table S2. LHWs competency and skills to provide maternal health care, Table S3. Administrative and logistic support available to LHW to provide maternal care. (DOC 44 kb) [file 12978_2016_215_MOESM1_ESM.doc]

Table S1 Baseline attributes of health care providers

| **Type of health care provider** | **Average years of experience**  **(Min, Max)** | **Average number of pregnant women seen daily**  **(Min, Max)** | **Average number of women with pre-eclampsia seen in last year**  **(Min, Max)** |
| --- | --- | --- | --- |
| Lady Health Supervisors | 15  (8,17) | 3  (2,15) | 6  (1, 25) |
| Traditional Birth Attendants | 23  (5, 50) | 5  (5, 9) | 2  (0, 7) |
| Woman Medical Officers | 17  (1/2, 26) | 24  (2, 40) | 22  (0, 100) |

Table S2 LHWs competency and skills to provide maternal health care

| Questions | Responses  N=457 | | | | | |
| --- | --- | --- | --- | --- | --- | --- |
|  | Don’t want to comment  n (%) | Disagree strongly  n (%) | Disagree  n (%) | Undecided  n (%) | Agree  n (%) | Agree strongly  n (%) |
| Can recognize pregnancy symptoms/signs | 4 (0.9) | 1 (0.2) | 1 (0.2) | 6 (1.3) | 26 (5.7) | 419 (91.7) |
| Can recognize pregnancy related danger signs/symptoms of hypertension | 45 (10.7) | 14 (3.1) | 30 (6.6) | 33 (7.2) | 38 (8.3) | 293 (64.1) |
| Seizure is a danger sign of pregnancy | 19 (4.6) | 2 (0.4) | 4 (0.9) | 8 (1.8) | 36 (7.9) | 386 (84.5) |
| Bleeding is a danger sign of pregnancy | 8 (2.2) | 2 (0.4) | 11 (2.4) | 9 (2) | 43 (9.4) | 382 (83.6) |
| Can check blood pressure | 161 (37.4) | 52 (11.4) | 52 (11.4) | 29 (6.3) | 22 (4.8) | 131 (28.7) |
| Can administer intramuscular injection | 33 (7.2) | 11 (2.4) | 6 (1.3) | 7 (1.5) | 27 (5.9) | 371 (81.2) |
| Can refer pregnant women to tertiary level hospital | 51 (11.2) | 7 (1.5) | 11 (2.4) | 7 (1.5) | 15 (3.3) | 361 (79.0) |
| Acceptance of referral by pregnant women | 15 (3.3) | 4 (0.9) | 2(0.4) | 3(0.7) | 25(5.5) | 407 (89.1) |

Table S3. Administrative and logistic support available to LHW to provide maternal care

| Questions | Responses  N=457 | | | | | |
| --- | --- | --- | --- | --- | --- | --- |
|  | Don’t want to comment  n (%) | Disagree strongly  n (%) | Disagree  n (%) | Undecided  n (%) | Agree  n (%) | Agree strongly  n (%) |
| Received trainings to identify pregnancy complications | 120 (26.3) | 61 (13.3) | 51(11.2) | 21 (4.6) | 7 (1.5) | 196 (42.9) |
| Received trainings to refer /manage complications of pregnancy | 106 (23.2) | 32 (7.0) | 40 (8.8) | 20 (4.4) | 21 (4.6) | 233 (51.0) |
| Have BP apparatus | 244 (53.4) | 93 (20.4) | 55 (12.0) | 16 (3.5) | 8 (1.8) | 30 (6.6) |
| Have weight machine | 156 (34.1) | 52 (11.4) | 33 (7.2) | 11 (2.4) | 9 (2.0) | 184 (40.3) |
| Have urine dipstick | 264 (57.8) | 86 (18.8) | 55 (12.0) | 19 (4.2) | 7 (1.5) | 15 (3.3) |
