# Supplementary material for: Health care provider knowledge and routine management of pre-eclampsia in Pakistan
Source: Reprod Health. 2016 Sep 30;13(Suppl 2):104. doi: 10.1186/s12978-016-0215-z (PMC5056497; doi:10.1186/s12978-016-0215-z)
Supplement: Additional file 2: — Review reports. (PDF 431 kb) [file 12978_2016_215_MOESM2_ESM.pdf]

Referee's comments to the authors– this sheet WILL be seen by the author(s) and published with the article

Title	Health care provider knowledge and routine management of pre-eclampsia in Pakistan
Author(s)	Sana Sheikh, Rahat Najam Qureshi, Asif Raza Khowaja, Rehana Salam, Marianne Vidler, Diane Sawchuck, Peter von Dadelszen, Shujat Zaidi, Zulfiqar Bhutta and the CLIP Working Group
Referee's name	Maryam Bigdeli

When assessing the work, please consider the following points, where applicable:

1. Is the question posed by the authors new and well defined? No, this not a new question and has been answered in previous publications, including in Pakistan
2. Are the methods appropriate and well described, and are sufficient details provided to replicate the work?
3. Are the data sound and well controlled? No, methods section needs more work.
4. Does the manuscript adhere to the relevant standards for reporting and data deposition? Not sure
5. Are the discussion and conclusions well balanced and adequately supported by the data? No
6. Do the title and abstract accurately convey what has been found? Yes
7. Is the writing acceptable? Yes

Please make your report as constructive and detailed as possible in your comments so that authors have the opportunity to overcome any serious deficiencies that you find and please also divide your comments into the following categories:

- Major Compulsory Revisions (which the author must respond to before a decision on publication can be reached)
- Minor Essential Revisions (such as missing labels on figures, or the wrong use of a term, which the author can be trusted to correct)
- Discretionary Revisions (which are recommendations for improvement but which the author can choose to ignore)

Where possible please supply references to substantiate your comments.

When referring to the manuscript please provide specific page and paragraph citations where appropriate.

(continue on the next sheet)

General comments:

This is an acceptable paper but does require more work on methods and presentation of results. I also struggle to find its added value compared to existing literature on pre-eclampsia (and in Pakistan)

Major compulsory revisions:

1. I struggle to understand the quantitative component: why would you use a Likert scale if you aggregate the data and finally report a binary response (positive or negative). How many categories did you use for your Likert scale and why didn't you exploit the Likert scale results in full? Responding to a Likert scale is not easy, respondents have to ponder their answers and moreover, you have applied this method to LHWs who have lower levels of education. I therefore do not understand why you use a sophisticated method to then simplify the results.
2. The quantitative results reported are minimalistic in regard of the number of respondents to the self-administered questionnaire and the efforts undergone to identify respondents in the community.
3. We also need a bit more data on your qualitative study sample and methods. Who were the 26 interviewees? Why 10 focus groups? Who participated in the FGDs? Why use both methods?
4. Also KII and focus group discussion transcripts should not be analyzed in the same way: it seems you handled the 2 types of transcripts in a similar fashion. However, you should consider that KII reflect individual responses while FGDs reflect group responses (in which you should analyze agreements and disagreements)
5. A mixed methods approach combining quantitative data analysis and qualitative data should aim at combining the sources and triangulating. Here you present the two sources of data one after the other and without link.
6. Key references on the topic are missing in your discussion:

Aaserud, M., Lewin, S., Innvaer, S., Paulsen, E. J., Dahlgren, A. T., Trommald, M., … Oxman, A. D. (2005). Translating research into policy and practice in developing countries: a case study of magnesium sulphate for pre-eclampsia. *BMC Health Services Research*, *5*, 68. http://doi.org/10.1186/1472-6963-5-68

Sevene, E., Lewin, S., Mariano, A., Woelk, G., Oxman, a D., Matinhure, S., … Daniels, K. (2005). System and market failures: the unavailability of magnesium sulphate for the treatment of eclampsia and pre-eclampsia in Mozambique and Zimbabwe. *BMJ*, *331*(7519), 765–9. http://doi.org/10.1136/bmj.331.7519.765

Duley, L., Gulmezoglu, A., Henderson/Smart, D., & Chou, D. (2010). Magnesium sulphate and other anticonvulsants for women with pre-eclampsia (Review). *Cochrane Database of Systematic Reviews*, (11), CD000025. http://doi.org/10.1002/14651858.CD000025.pub2.Copyright

Ridge, A. L., Bero, L. A., & Hill, S. R. (2010). Identifying barriers to the availability and use of Magnesium Sulphate Injection in resource poor countries : A case study in Zambia. *BMC Health Services Research*, *10*(1), 340. http://doi.org/10.1186/1472-6963-10-340

Lumbiganon, P., Metin Gulmezoglu, A., Piaggio, G., Langer, A., & Grimshaw, J. (2007). Magnesium suflate is not used for pre-eclampsia and eclampsia in Mexico and Thailand as much as it should be. *Bulletin of the World Health Organization*, *85*(10), 763–767. http://doi.org/10.2471/BLT.06.037911

7. You refer to Bigdeli et al in your discussion. Bigdeli et al conclude that training of health professionals on pre-eclampsia, eclampsia and use of magnesium sulfate in the context of Pakistan is far from sufficient and a systemic approach is needed that combines this training with multiple system levels interventions. Ridge et al conclude to the same in other settings than Pakistan. In view of that, I find your conclusions and recommendations very thin and with limited added value.

Minor essential revisions:

Discretionary revisions:

Referee's comments to the authors– this sheet WILL be seen by the author(s) and published with the article

Title	Health care provider knowledge and routine management of pre-eclampsia in Pakistan
Author(s)	Sana Sheikh, Rahat Najam Qureshi, Asif Raza Khowaja, Rehana Salam, Marianne Vidler, Diane Sawchuck, Peter von Dadelszen, Shujat Zaidi, Zulfiqar Bhutta and the CLIP Working Group
Referee's name	Nageena Mahmood

When assessing the work, please consider the following points, where applicable:

1. Is the question posed by the authors new and well defined?
2. Are the methods appropriate and well described, and are sufficient details provided to replicate the work?
3. Are the data sound and well controlled?
4. Does the manuscript adhere to the relevant standards for reporting and data deposition?
5. Are the discussion and conclusions well balanced and adequately supported by the data?
6. Do the title and abstract accurately convey what has been found?
7. Is the writing acceptable?

Please make your report as constructive and detailed as possible in your comments so that authors have the opportunity to overcome any serious deficiencies that you find and please also divide your comments into the following categories:

- Major Compulsory Revisions (which the author must respond to before a decision on publication can be reached)
- Minor Essential Revisions (such as missing labels on figures, or the wrong use of a term, which the author can be trusted to correct)
- Discretionary Revisions (which are recommendations for improvement but which the author can choose to ignore)

Where possible please supply references to substantiate your comments.

When referring to the manuscript please provide specific page and paragraph citations where appropriate.

General comments: This study highlights knowledge deficiencies amongst the various health care providers a pregnant woman in rural Sindh may come across, thus paving the way for recommendations and guideline e development to facilitate the management of ore eclampsia in this setting.

Major compulsory revisions:

Minor essential revisions:

Discretionary revisions:

(continue on the next sheet)

Supplement Editor's comments

Please in the first sentence of the Discussion section make an statement of principal findings of the study.
